# Dengue-1 virus and vector competence of *Aedes aegypti* (Diptera: Culicidae) populations from New Caledonia

**DOI:** 10.1186/s13071-017-2319-x

**Published:** 2017-08-09

**Authors:** Elodie Calvez, Laurent Guillaumot, Dominique Girault, Vaea Richard, Olivia O’Connor, Tuterarii Paoaafaite, Magali Teurlai, Nicolas Pocquet, Van-Mai Cao-Lormeau, Myrielle Dupont-Rouzeyrol

**Affiliations:** 1 Institut Pasteur de Nouvelle-Calédonie, URE-Dengue et autres Arboviroses, Réseau International Institut Pasteur, Nouméa, New Caledonia; 2 Institut Pasteur de Nouvelle-Calédonie, URE-Entomologie Médicale, Réseau International Institut Pasteur, Nouméa, New Caledonia; 3 Institut Louis Malardé, Papeete, French Polynesia; 4 Institut Pasteur de Nouvelle-Calédonie, URE-Epidémiologie des Maladies Infectieuses, Réseau International Institut Pasteur, Nouméa, New Caledonia

**Keywords:** Dengue virus (DENV), *Aedes aegypti*, New Caledonia, Pacific region, Vector competence

## Abstract

**Background:**

Dengue virus (DENV) is the arbovirus with the highest incidence in New Caledonia and in the South Pacific region. In 2012–2014, a major DENV-1 outbreak occurred in New Caledonia. The only known vector of DENV in New Caledonia is *Aedes aegypti* but no study has yet evaluated the competence of New Caledonia *Ae*. *aegypti* populations to transmit DENV. This study compared the ability of field-collected *Ae*. *aegypti* from different locations in New Caledonia to transmit the DENV-1 responsible for the 2012–2014 outbreak. This study also aimed to compare the New Caledonia results with the vector competence of *Ae*. *aegypti* from French Polynesia as these two French countries have close links, including arbovirus circulation.

**Methods:**

Three wild *Ae*. *aegypti* populations were collected in New Caledonia and one in French Polynesia. Female mosquitoes were orally exposed to DENV-1 (10^6^ FFU/ml). Mosquito bodies (thorax and abdomen), heads and saliva were analyzed to measure infection, dissemination, transmission rates and transmission efficiency, at 7, 14 and 21 days post-infection (dpi), respectively.

**Results:**

DENV-1 infection rates were heterogeneous, but dissemination rates were high and homogenous among the three *Ae*. *aegypti* populations from New Caledonia. Despite this high DENV-1 dissemination rate, the transmission rate, and therefore the transmission efficiency, observed were low. *Aedes aegypti* population from New Caledonia was less susceptible to infection and had lower ability to transmit DENV-1 than *Ae*. *aegypti* populations from French Polynesia.

**Conclusion:**

This study suggests that even if susceptible to infection, the New Caledonian *Ae*. *aegypti* populations were moderately competent vectors for DENV-1 strain from the 2012–2014 outbreak. These results strongly suggest that other factors might have contributed to the spread of this DENV-1 strain in New Caledonia and in the Pacific region.

**Electronic supplementary material:**

The online version of this article (doi:10.1186/s13071-017-2319-x) contains supplementary material, which is available to authorized users.

## Background

Dengue fever is one of the most prevalent human vector-borne diseases in tropical and subtropical countries. A recent study estimates that 390 million dengue fever infections occur every year worldwide [1]. Dengue virus (DENV) is a single-stranded, positive-sense RNA virus of the genus *Flavivirus*, separated in four serotypes (DENV-1 to DENV-4) themselves divided into genotypes [2]. Infection by one of these serotypes confers specific and prolonged immunity against that serotype only [3]. DENV is transmitted to humans through the bite of mosquitoes of the genus *Aedes*, subgenus *Stegomyia*.

Unlike countries in south-east Asia and Latin America where dengue is hyperendemic, dengue in the Pacific region is characterized by intermittent epidemics of varying severity. During the second half of the twentieth century, dengue epidemics in the Pacific were mainly caused by a single serotype/genotype introduced from another, hyperendemic country. The epidemiology of dengue, however, is heterogeneous; Small Pacific Island Countries and Territories (PICTs) are affected by DENV transmission for a few months only, while larger PICTs, like New Caledonia (NC) or French Polynesia (FP), may experience active circulation of a single serotype/genotype for several years until the emergence of a new epidemic viral strain [4–8].

In the Pacific region, known vectors of DENV are *Aedes aegypti*, *Aedes albopictus*, *Aedes polynesiensis* and other local species [9]; *Ae*. *aegypti* is present throughout the region with the exception of a few isolated islands [10]. The introduction of *Ae*. *aegypti* in the Pacific is relatively recent, starting in the late nineteenth century [9]. Previous studies found differences in the genetic structure of Pacific populations of *Ae*. *aegypti* [11–13], with significant genetic differentiation between *Ae*. *aegypti* from NC compared to populations from other PICTs [13]. In NC, a lower genetic differentiation was observed between the mosquitoes from the main island (Nouméa and Poindimié sampling sites) and the mosquitoes from Ouvéa, a smaller, distant island [13]. This genetic differentiation may impact the vector capacity of the mosquito [14, 15]. Indeed, other studies showed that vector competence for DENV may be linked to the genetic background of the vector, such as genes related to the midgut escape barrier, notably the Quantitative Trait Loci (QTL) on the chromosome III [16–18].

New Caledonia is a French territory located in the tropical zone of the South Pacific Ocean. Dengue transmission in NC occurs mainly during the hot-and-rainy season (December to May). Epidemics have a 3–4 years cyclical pattern [4]. Dengue epidemics usually last 2 years, with two peaks occurring during the two consecutive hot-and-rainy seasons and few cases reported during the interepidemic cool season. Since World War II, NC has experienced 13 epidemics, caused by all four DENV serotypes. Most of these epidemics occurred after the introduction of a new viral strain either directly from south-east Asia or after emerging in another PICT [19]. When a new DENV serotype is introduced in NC, the previously circulating serotype is usually replaced within a few months. This pattern, however, is evolving. After causing severe epidemics in 2003–2004 and again in 2008–2009, DENV-1 unexpectedly re-emerged in 2012–2014 and caused the largest outbreak ever reported in NC [4]. The spatial distribution of dengue cases on the main island showed that the East coast was more affected with higher reported dengue incidence rates than the West coast [20]. *Aedes aegypti* is the only mosquito species recognized as being able to transmit DENV in NC but, to date, no study has evaluated its competence to transmit.

In the present study, we aimed to test the susceptibility of *Ae*. *aegypti* populations from different locations in NC to DENV. We collected three *Ae*. *aegypti* populations based on DENV cases distribution in NC and performed vector competence assays with a DENV-1 strain isolated during the 2012–2014 outbreak. There are close economics and population exchanges between New Caledonia and French Polynesia and previous work highlighted similarities in arbovirus transmission between these two territories [21–23]. In the present study, we therefore compared the vector competence obtained for *Ae*. *aegypti* from Nouméa, the largest city in New Caledonia, with the vector competence of *Ae*. *aegypti* from Papeete, the largest city in French Polynesia.

## Methods

### Mosquitoes

**Fig. 1:**
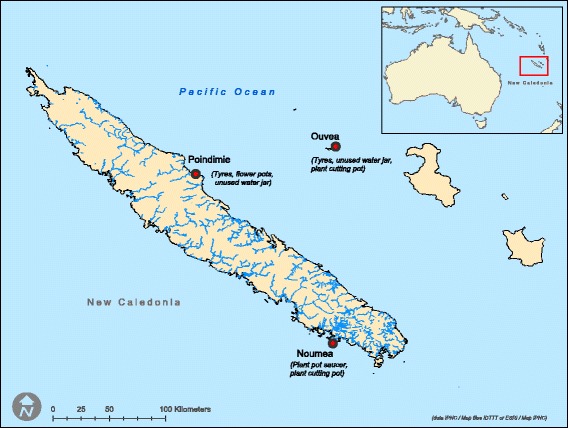
Map showing *Ae*. *aegypti* sampling sites in New Caledonia, 2015. The three sample sites are represented by *red dots*. Breeding sites are indicated in parentheses

Mosquitoes were sampled at immature stages (larvae and pupae) in three sites in New Caledonia (Nouméa, Poindimié and Ouvéa; Fig. 1) [13] and in one site in French Polynesia (Papeete, Tahiti Island). Larvae and pupae were reared to adult forms which were maintained at 28 °C and 80% humidity, a 12:12 h light-dark cycle and fed with a 10% sucrose solution. Females were blood-fed several times with guinea pig blood to obtain F1-generation eggs. For all infection assays, F1-eggs were hatched and adults were maintained as described above.

### Viral strain

The viral strain used in this study was a DENV-1 genotype I isolated from a patient in New Caledonia in 2014 (GenBank KY553285). The virus stock was obtained after five passages of 5 days incubation at 28 °C on *Ae*. *albopictus* C6/36 cells in Leibovitz L15 medium (Sigma-Aldrich, Saint-Louis, Missouri, USA) supplemented with 2% fetal bovine serum (FBS; Gibco BRL, Paisley, Scotland, UK) and 10% of tryptose phosphate (Gibco BRL, Paisley, Scotland, UK). The titration of the viral stock was performed using immunofluorescent assays with the anti-dengue virus complex antibody, clone D3-2H2–9-21 (Millipore, Billerica, Massachusetts, USA) and the Alexa Fluor 488 goat anti-mouse IgG secondary antibody (Life technologies, Eugene, Oregon, USA) and the results calculated in FFU/ml (Focus Forming Unit).

### Mosquito oral infections

Five to seven day-old, never blood-fed F1-females were starved for 24 h before infection. They were allowed to take an infectious blood meal through an artificial system with pig intestine membrane stretched over the Hemotek system (Discovery Workshops, Accrington, UK), containing a mix (2:1) of rabbit washed erythrocytes and viral suspension supplemented with adenosine triphosphate (Sigma-Aldrich, Saint-Louis, Missouri, USA) at 5 mM. The female mosquitoes were allowed access to the blood meal maintained at 37 °C for 20 min. The average concentration of DENV-1 in blood meals was 10^6^ FFU/ml. After blood-feeding, fully engorged females were transferred into new containers and maintained at 28 °C and 80% humidity with a 12:12 h light-dark cycle and ad libitum access to 10% sucrose.

### Dissemination and transmission analyses

At 7, 14 and 21 days post-infection (dpi) for NC populations and at 21 dpi for the FP population from Papeete (FP-Papeete), 30 mosquito females (except for Nouméa and Papeete at 21 dpi with 25 and 17 mosquitoes, respectively) were randomly collected and cold-anesthetized. Legs and wings of each sampled mosquito were removed and the proboscis was inserted for 30 min into a filter tip ART (Thermo Scientific, San Diego, USA) containing 5 μl of FBS for salivation. The 5 μl harvested were added to 45 μl of L-15 medium and preserved at -80 °C until analysis. The body and the head of each mosquito were placed in separate tubes and stored at -80 °C until use.

To determine infection and dissemination characteristic of the four mosquito populations, the head and the body of each mosquito were mechanically ground with ceramic beads (Roche, Auckland, New Zealand) in 350 μl of L-15 medium supplemented with 5% FBS, 10% tryptose phosphate and antibiotics/antifungals (100 units/ml of penicillin, 0.1 mg/ml of streptomycin and 0.25 μg/ml amphotericin B). Lysis was performed three times during 30 s at 3000 rpm and samples were then centrifuged at 5000 rpm for 5 min. For each sample, 140 μl of supernatant were used for RNA extraction with the QIAamp Viral RNA Mini Kit (Qiagen, Hilden, Germany). Viral detection was performed using real time reverse transcription polymerase chain reaction (RT-PCR) by LightCycler 480 II (Roche, Auckland, New Zealand) and previously published primers and probe [24] and the SuperScript III One-Step RT-PCR System with Platinum Taq DNA Polymerase (Invitrogen, Carlsbad, California, USA).

To determine mosquitoes’ transmission ability, saliva samples were inoculated onto *Ae*. *albopictus* C6/36 cells in 96-well plates and incubated at 28 °C for 5 days. DENV infective particles were detected was achieved by immunofluorescent assays as described above.

### Data analysis

The infection rate (number of positive bodies divided by the total number of mosquitoes tested), dissemination rate (number of infected heads divided by the number of infected bodies), transmission rate (number of infected saliva divided by the number of infected heads) and transmission efficiency (number of infected saliva divided by the total number of mosquitoes tested) were calculated for each *Ae*. *aegypti* population at each dpi. Data were statistically compared using Chi-square test or Fisher’s exact test using R software (v. 3.3.1 [25]), considering *P*-values > 0.05 as non-significant.

## Results

### Fluctuant infection rate in NC *Ae. aegypti* population

**Fig. 2:**
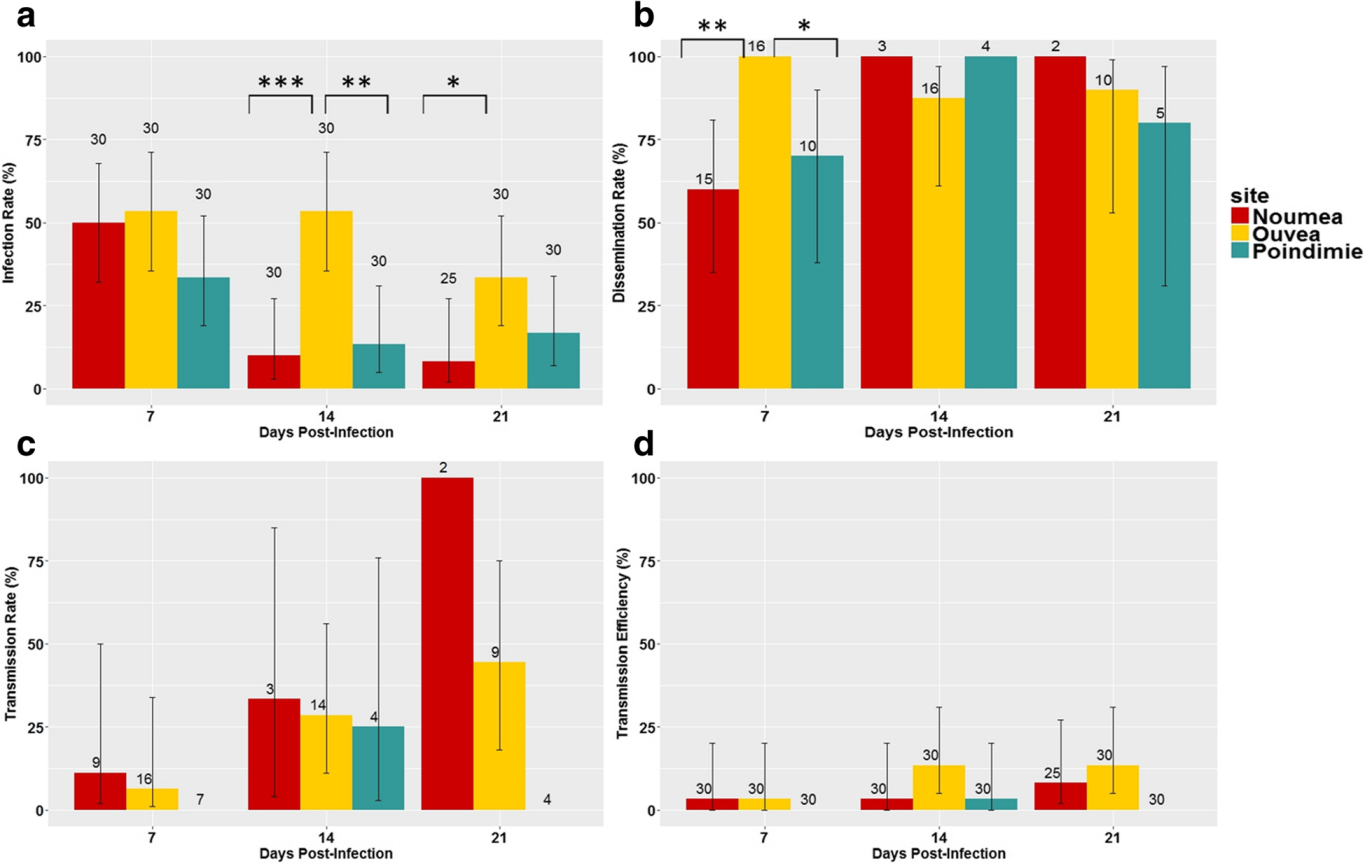
DENV vector competence results for NC mosquitoes (7, 14 and 21 dpi). DENV-1 infection rates (**a**), dissemination rates (**b**), transmission rates (**c**) and transmission efficiencies (**d**). Error bars represent 95% confidence intervals. Numbers of mosquitoes tested in each condition are indicated above each barplot. Significant differences are indicated by *asterisks* (*P < 0.05; **P < 0.01; ***P < 0.001)

At 7 dpi (Fig. 2a, Additional file 1: Table S1), infection rates ranged from 33 to 53% for the three NC populations with no significant difference measured. From 14 dpi, infection rates decreased significantly in *Ae*. *aegypti* mosquitoes from Poindimié (Fisher’s exact test: *P* = 0.0022 at 14 dpi) and Nouméa (Fisher’s exact test: *P* = 0.0006 and 0.0463 at 14 and 21 dpi, respectively) compared to the Ouvéa population.

### High dissemination but low transmission in NC *Ae. aegypti* populations

Dissemination rates ranged from 60 to 100% at 7dpi for all NC mosquitoes tested, with Ouvéa mosquitoes showing significantly higher dissemination rates when compared to Poindimié (Fisher’s exact test: *P* = 0.046) and Nouméa (Fisher’s exact test: *P* = 0.007) (Fig. 2b, Additional file 1: Table S1). Although the number of infected mosquitoes obtained for Nouméa and Poindimié were low after 14 dpi, the dissemination rate seems more homogenous among the three NC populations (80–100% of dissemination).

Infectious DENV-1 particles were detected in the saliva of tested *Ae*. *aegypti* mosquitoes from Nouméa and Ouvéa as early as 7 dpi and at 14 dpi for Poindimié (Fig. 2c, Additional file 1: Table S1). The transmission efficiencies of the three NC populations ranged from 3 to 13% (Fig. 2d, Additional file 1: Table S1).

### Lower competence of NC-Nouméa *Ae. aegypti* for DENV-1 compared to PF-Papeete *Ae. aegypti*

**Fig. 3:**
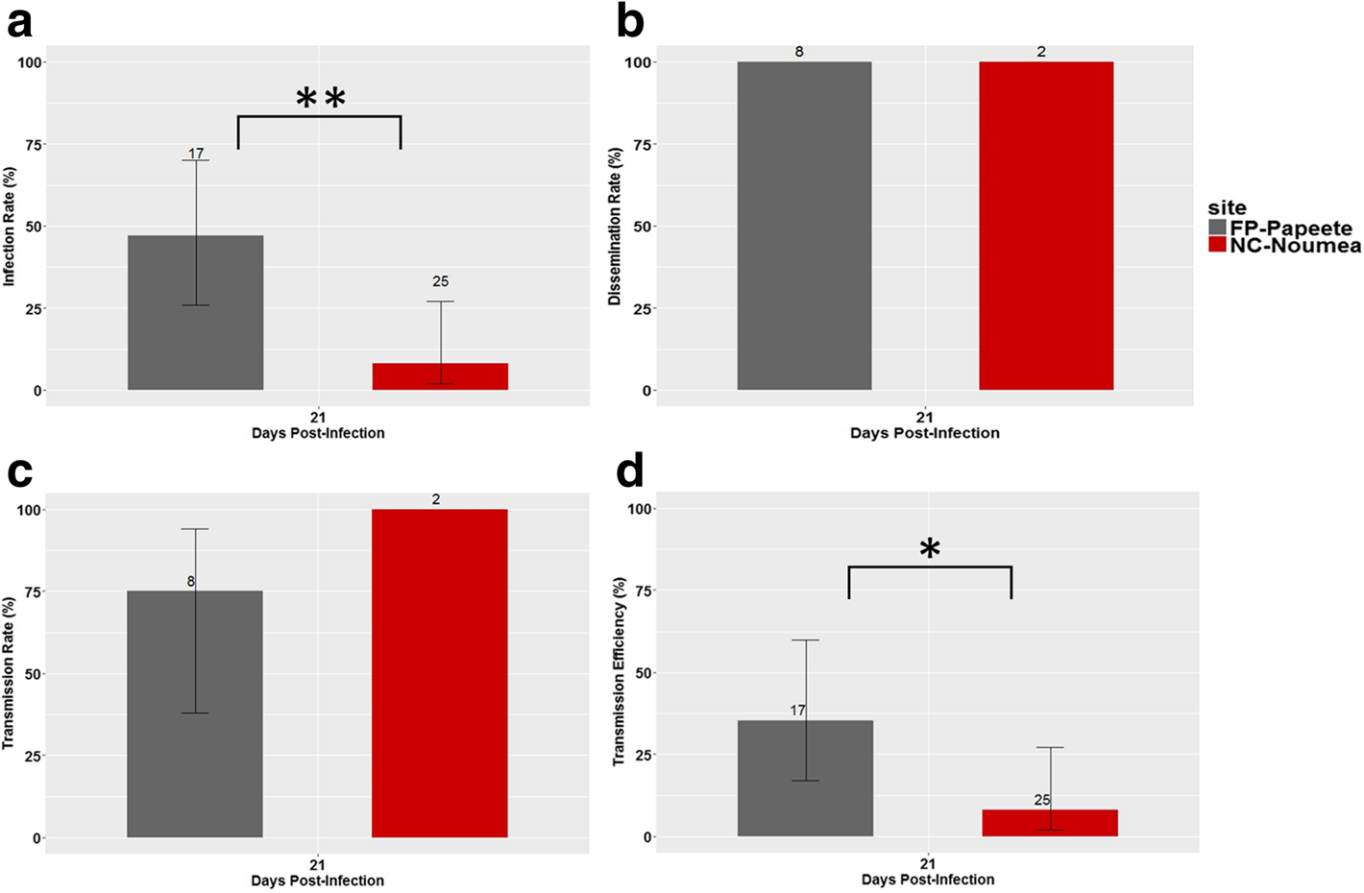
Comparison between *Ae*. *aegypti* population from Nouméa (NC) and Papeete (FP) (21 dpi). Comparison of DENV-1 infection rates (**a**), dissemination rates (**b**), transmission rates (**c**) and transmission efficiencies (**d**). *Error bars* represent 95% confidence intervals. Numbers of mosquitoes tested are indicated above each barplot. Significant differences are indicated by asterisks (*P < 0.05; **P < 0.01; ***P < 0.001)

Because NC and FP are both French territories in the Pacific region, they have long and close ties, including arbovirus circulation. We therefore compared the vector competence of both *Ae*. *aegypti* collected in the two capital cities, (Nouméa and Papeete). Regarding FP-Papeete mosquitoes, 47% were infected at 21 dpi compared to 8% observed in the NC-Nouméa population (Fisher’s exact test: *P* = 0.008). In both Nouméa and Papeete populations, dissemination rate observed among the infected individuals reached 100% (Fig. 3, Additional file 1: Table S1). The DENV-1 transmission rate calculated exclusively among the mosquitoes in which the virus had disseminated was 75% (*n* = 8) for the FP-Papeete population. The transmission efficiency differed significantly between the FP-Papeete and NC-Nouméa populations, at 35% (*n* = 17) and 8% (*n* = 25), respectively (Fisher’s exact test: *P* = 0.045).

## Discussion

Recent studies have characterized the vector competence of several populations of *Ae*. *aegypti* from the Pacific region for arboviruses, especially chikungunya and Zika viruses [26–28]. The present study is the first to describe and compare the vector competence of three *Ae*. *aegypti* populations from NC for dengue virus serotype 1.

DENV-1 has been the major circulating serotype in the Pacific region over the past decade [4, 19]. The DENV-1 strain used in this study belongs to the Asian genotype and was isolated in 2014 during the largest dengue fever outbreak reported in NC [4]. This genotype, introduced in NC in 2012, subsequently spread to other PICTs (Vanuatu and FP) and is still circulating in NC and FP (O. O’Connor, personal communication). This ongoing circulation suggests efficient transmission of this DENV-1 strain by local vectors.

Our results confirm New Caledonian *Ae*. *aegypti* populations’ ability to be infected by and subsequently to disseminate DENV-1. Infection rates differ significantly between the Ouvéa population and the Nouméa and Poindimié populations, related to a decrease overtime for the latter two. This decrease in susceptibility to infection in these mosquitoes suggests that either: 1) survival rate could be lower in the infected than in the non-infected mosquitoes; or 2) *Ae*. *aegypti* from Nouméa and Poindimé could be more resistant to infection by this DENV-1 stain than mosquitoes from Ouvéa. The three NC populations were collected from different localities, but their environments may have been different [13]. The resident microbiota, which directly depends on the local ecosystem and lives in the breeding site, are known to influence the adult mosquito microbiome. Interestingly, the bacteria from that microbiome can modulate mosquitoes’ viral susceptibility [29, 30]. Regulation of host immunity is one possible mechanism by which the microbiome could influence mosquito susceptibility to regulate of innate immune responses, especially via the RNA interference pathways and strongly alter the viral replication capacity [31, 32]. Moreover, the re-introduction of specific microbiome bacteria in the midgut of mosquitoes treated with antibiotics to remove their midgut flora has shown to decrease DENV infection rates, possibly through the activation of immune system factors [33]. One can therefore hypothesize that a microbiome-dependent up-regulation of NC mosquitoes’ immune responses could be the reason for our observed decrease in infection rates. As the microbiota in the breeding site can impact the adult mosquito microbiome [34], differences in the ecosystem may influence the mosquitoes’ innate immune response [33, 35, 36] and the consequent vector competence. The microbiota could play a role in the modulation of DENV infection through a possibly basal-level stimulation of the antiviral immune system of mosquitoes [37]. Several innate immune pathways are activated during arbovirus infection, especially the Toll pathway, the Jak-STAT pathway and the mosquito RNAi. All these pathways are known to control arbovirus infection and transmission in the mosquitoes [31, 37–41]. Thus, the up-regulation of these pathways in time could explain the lower DENV infection rate observed in NC mosquitoes.

Despite reasonable numbers of mosquitoes collected at each dpi (25–30 mosquitoes), unexpectedly low infection rates unfortunately resulted in small sample sizes for the estimation of dissemination and transmission rates. Our study, however, provides interesting data on dissemination and transmission of DENV-1 in NC mosquitoes. Although infection rates were modest, the dissemination of the virus in infected mosquitoes was high in the three NC populations. The dissemination rates obtained among NC populations were high and relatively homogenous, except at 7 dpi for which the population from Ouvéa had a higher dissemination rate. Transmission efficiencies were also similar between the three populations and they were surprisingly low, as observed in previous studies [42–44].

New Caledonia and French Polynesia are two overseas territories administrated by France and, consequently, have long-standing ties and close exchanges (e.g. economics, populations and travel). Previous work has highlighted similarities in arbovirus transmission between these two territories and showen that the same DENV-1 strain circulates in both territories [4, 19, 22]. We compared the ability of the *Ae*. *aegypti* population from Nouméa, the capital city of New Caledonia, with the ability of *Ae*. *aegypti* from Papeete, the capital city of French Polynesia, to transmit this DENV-1 strain. The results clearly demonstrate a lower susceptibility to infection and lower transmission efficiency in NC mosquitoes.

Several factors may influence vector competence of *Ae*. *aegypti*, notably host viremia, the genetic background of the *Ae*. *aegypti* populations tested and/or the virulence of the virus strain used [14, 15, 45]. As shown in previous studies [46, 47], vector competence is a dose-response phenomenon. In this study, all mosquitoes were fed with a blood meal at the average concentration of 10^6^ FFU/ml. As the same DENV-1 strain was used to infect these four mosquito populations, the vector’s genetic characteristics are probably an important parameter in the observed difference in viral transmission. Variation in vector competence of *Ae*. *aegypti* has previously been observed between populations from different countries, but also within a country [42–44, 48]. The mosquito populations tested here were collected in the same sampling sites used in a recently published study, which showed genetic differences between the Ouvéa *Ae*. *aegypti* population and the Nouméa and Poindimié populations, and between the Nouméa and the Papeete population [13].

The known vector for dengue virus in New Caledonia is *Ae*. *aegypti*. In FP and the rest of the South Pacific region, however, other vectors are present, especially *Ae*. *polynesiensis* and *Ae*. *albopictus* [9, 10]. Although the *Ae*. *aegypti* populations tested were susceptible to infection in the present study, they were unexpectedly low competence vectors for the strain of DENV-1 circulating since 2012. The prolonged circulation of DENV-1 in NC may therefore not be due solely to the vector-virus interaction, and highlights the importance of several other factors, such as vector density, mosquito lifespan, the number of susceptible individuals in the human population as well as frequency and intensity of host-vector contact. This work underscores the importance of maintaining both arboviruses and vector surveillance networks in the Pacific and the need to develop innovative and targeted vector control strategies in the region to prevent expansion of outbreaks [49, 50].

## Conclusion

In the context of globalization of trade and travel, and as shown with chikungunya or the recent Zika outbreak that spread from the Pacific region to the Americas [51], improving our knowledge on Pacific region vectors and arboviruses’ circulation is of major importance. This study describes, for the first time, the vector competence of several populations of *Ae*. *aegypti* from NC for the DENV-1 strain currently circulating in this country and the rest of the Pacific. Studies are now needed to determine the vector competence of NC *Ae*. *aegypti* for other serotypes/genotypes of DENV and for other arboviruses circulating in the Pacific, to guide risk assessment strategies in NC and the rest of the Pacific region.

## Additional file

Additional file 1: Table S1. Infection, dissemination, transmission rates and transmission efficiency at 7, 14 and 21 days post-infection (dpi). (DOCX 13 kb)

## Abbreviations

DENV: Dengue virus
dpi: day post-infection
FBS: Fetal bovine serum
FFU: Focus Forming Unit
FP: French Polynesia
NC: New Caledonia
PICTs: Pacific Island Countries and Territories
QTL: Quantitative Trait Loci
RT-PCR: Reverse Transcription Polymerase Chain Reaction
