# Supplementary material for: Dengue-1 virus and vector competence of Aedes aegypti (Diptera: Culicidae) populations from New Caledonia
Source: Parasit Vectors. 2017 Aug 9;10:381. doi: 10.1186/s13071-017-2319-x (PMC5551013; doi:10.1186/s13071-017-2319-x)
Supplement: Additional file 1: Table S1. — Infection, dissemination, transmission rates and transmission efficiency at 7, 14 and 21 days post-infection (dpi). (DOCX 13 kb) [file 13071_2017_2319_MOESM1_ESM.docx]

**Table S1:** Infection, dissemination, transmission rates and transmission efficiency at 7, 14 and 21 days post-infection (dpi)

|  | | 7 dpi | 14 dpi | 21dpi |
| --- | --- | --- | --- | --- |
| Number of infected bodies / number of mosquitoes tested  (% of infection) | NC -Nouméa | 15/30 (50%) | 3/30 (10%) | 2/25 (8%) |
|  | NC - Ouvéa | 16/30 (53%) | 16/30 (53%) | 10/30 (33%) |
|  | NC - Poindimié | 10/30 (33%) | 4/30 (13%) | 5/30 (17%) |
|  | FP - Papeete | – | – | 8/17 (47%) |
| Number of infected heads / number of infected bodies  (% of dissemination) | NC -Nouméa | 9/15 (60%) | 3/3 (100%) | 2/2 (100%) |
|  | NC - Ouvéa | 16/16 (100%) | 14/16 (87%) | 9/10 (90%) |
|  | NC - Poindimié | 7/10 (70%) | 4/4 (100%) | 4/5 (80%) |
|  | FP - Papeete | – | – | 8/8 (100%) |
| Number of infected saliva / number of infected heads  (% of transmission) | NC -Nouméa | 1/9 (11%) | 1/3 (33%) | 2/2 (100%) |
|  | NC - Ouvéa | 1/16 (6%) | 4/14 (29%) | 4/9 (44%) |
|  | NC - Poindimié | 0/7 (0%) | 1/4 (25%) | 0/4 (0%) |
|  | FP - Papeete | – | – | 6/8 (75%) |
| Number of infected saliva / number of mosquitoes tested  (% of efficiency) | NC -Nouméa | 1/30 (3%) | 1/30 (3%) | 2/25 (8%) |
|  | NC - Ouvéa | 1/30 (3%) | 4/30 (13%) | 4/30 (13%) |
|  | NC - Poindimié | 0/30 (0%) | 1/30 (3%) | 0/30 (0%) |
|  | FP - Papeete | – | – | 6/17 (35%) |
